# Direct costs of integrated procedures of conventional hemodialysis performed by nursing professionals^1^

**DOI:** 10.1590/1518-8345.1812.2944

**Published:** 2018-07-16

**Authors:** Antônio Fernandes Costa Lima

**Affiliations:** 2 PhD, Associate Professor, Escola de Enfermagem, Universidade de São Paulo, São Paulo, SP, Brasil.

**Keywords:** Hemodialysis Units, Hospital, Nephrology Nursing, Costs and Cost Analysis, Cost Control

## Abstract

**Objective:**

to analyze the mean direct cost of the constituent procedures of conventional hemodialysis, performed in three public teaching and research hospitals.

**Method:**

quantitative, exploratory-descriptive study, of the multiple case study type. The mean direct cost was calculated by multiplying the time (timed) spent by nursing professionals, on the execution of procedures, by the unit cost of direct labor, added to the cost of materials and solutions/medications.

**Results:**

the total mean direct cost, in patients with an arteriovenous fistula corresponded to US$25.10 in hospital A, US$37.34 in hospital B and US$25.01 in hospital C, and in patients with a dual lumen catheter, US$32.07 in hospital A, US$40.58 in hospital B and US$30.35 in hospital C. The weighted mean values obtained were US$26.59 for hospital A, US$38.96 for hospital B and US$27.68 for hospital C. It was noted that the “installation and removal of hemodialysis fistula access” caused a significantly lower economic impact compared to “installation and removal of hemodialysis catheter access”.

**Conclusion:**

with the knowledge developed it will be possible to support hospital managers, technical managers and nursing professionals in the decision making process, with a view to the rational allocation of the necessary inputs for the performance of conventional hemodialysis.

## Introduction

The worldwide increase in the prevalence of Chronic Kidney Disease (CKD) has required the adoption of one of the available types of dialysis - hemodialysis, peritoneal dialysis, renal transplantation, significantly burdening the finite health budgets^1^ ^-^ ^2^. In Brazil, government agencies have been preoccupied with the costs of renal replacement therapies, since care for patients with CKD, usually performed at dialysis centers of hospital organizations, is one of the main areas that make up the high complexity of the Brazilian Nation Health System (SUS), consuming a large amount of financial resources^3^.

Hemodialysis represents the main type of dialysis adopted worldwide for the treatment, control and vital maintenance of patients with CKD^2^, which requires specialized care and advanced technology. Properly trained and highly qualified nurses and nursing technicians are responsible for the component procedures of conventional hemodialysis, the costs of which are unknown at dialysis centers and in hospital organizations. Consequently, the decision-making process related to the allocation efficiency of limited resources, the establishment of a rational basis to support the negotiation of adequate financial transfer, along with funding sources, and planning for future investments can be seriously compromised.

Increased demand for health services, high healthcare costs and limited resources strongly pressure health organizations to become efficient, increase their productivity and minimize their spending. To that end, their care and management processes must be meticulously studied in order to align resources and actions^4^.

Considering these facts, the present study was carried out in order to analyze the mean direct cost of the integrated procedures of conventional hemodialysis performed by nursing professionals in three public teaching and research hospitals in the state of São Paulo.

## Method

This was a quantitative, exploratory-descriptive, multiple case type study^5^ conducted at the dialysis centers of three public teaching and research hospitals, after approval by the Research Ethics Committee of the proposing institution (Authorization No. 489.961 ) and co-participating institutions (Authorization No. 492.808, Authorization no. 555.641 and Co-participant Communiqué of 02/15/2014).

The dialysis centers of these hospitals, referred to as A, B and C, were selected due to presenting good nursing practices, together with the adequate technological structure and human resources, quantitative and qualitative, necessary for the care of patients with CKD. Prior meetings were held with the coordinators of the dialysis centers for the detailed presentation of the research project and they stated that, with the approval of the respective Research Ethics Committees, there would be no impediments to the release of the information for the costing of the procedures.

The sample size was calculated by a statistics professional, based on a 95% confidence interval and a tolerable statistical error of 10%, corresponding to at least 100 observations of each of the hemodialysis components studied at the three dialysis centers. Thus, the study sample consisted of 1,928 observations of procedures, such as “preparation of extracorporeal circuit for hemodialysis” (*n*=614), “installation of hemodialysis arteriovenous fistula or dual lumen catheter access” (*n*=657) and “removal of hemodialysis arteriovenous fistula or dual lumen catheter access” (*n*=657). Considering that the professionals empirically attribute higher costs to installation/removal of catheter access, even without knowing their costs, it was decided to present these procedures, separately, according to the access route used.

The intervening variables in the direct cost of the integrated hemodialysis procedures, as well as the relationship of these variables, were first established, with the total mean direct cost obtained by the sum of the mean costs^6^:

$$\bar{\;Cd_{HCD}}=\overset{j}{\underset{i=1}{\sum }}\bar{CP_{t}}$$ (1).

Considering that the procedures would consume different quantities of inputs, the total mean direct cost of each procedure was established composed of three parts: mean direct cost of materials , of solutions/medications and of labor :

$$\bar{C\left(P_{i}\right)}=\bar{C{\left(P_{i}\right)}_{mat}}+\bar{C{\left(P_{i}\right)}_{sol}}+\bar{C{\left(P_{i}\right)}_{mob}}$$ (2).

The mean direct cost of the materials was obtained by summing the mean costs of each of the materials consumed:

$$\bar{\;C{\left(P_{i}\right)}_{mat}}=\overset{n}{\underset{k=1}{\sum }}\bar{Cm_{k}}$$ (3).

The mean cost of each material was obtained from the product of the mean quantity of this material by its mean unit price:

$$\bar{Cm_{k}}=\bar{qm_{k}}\cdot \bar{Pmu_{k}}$$ (4).

Substituting equation 3 with 4 gave a more detailed equation for the mean direct cost of materials:

$$\bar{\;C{\left(P_{i}\right)}_{mat}}=\overset{n}{\underset{k=1}{\sum }}\;\left(\;\bar{q_{mk}}\;\cdot \bar{Pmu_{k}}\right)$$ (5).

The mean direct cost of the solutions/medications was obtained by summing the mean costs of solutions/medications consumed:

$$\bar{\;C{\left(P_{i}\right)}_{sol}}=\overset{n}{\underset{k=1}{\sum }}\bar{Cs_{k}}$$ (6).

The mean cost of each solution/medication was obtained from the product of the mean quantity of this solution/medication by its mean unit price :

$$\bar{Cs_{k}}=\bar{qs_{k}}\cdot \bar{Psu_{k}}$$ (7).

Substituting equation 6 with 7 gave a more detailed equation for the mean direct cost of solutions/medications:

$$\bar{\;C{\left(P_{i}\right)}_{sol}}=\overset{n}{\underset{k=1}{\sum }}\;\left(\;\bar{qs_{k}}\;\cdot \bar{Psu_{k}}\right)$$ (8).

The mean direct cost of labor was obtained by summing the mean costs of each professional category (nurses and nursing technicians) involved in the procedures, as follows:

$$\bar{\;C{\left(P_{i}\right)}_{mob}}=\overset{n}{\underset{c=1}{\sum }}\bar{Ch_{c}}$$ (9).

The mean cost of each professional category was obtained from the product of the mean time spent by each category on the procedures by the mean unit cost of labor:

$$\bar{Ch_{c}}=\bar{t_{c}}\cdot \bar{Su_{c}}$$ (10).

Substituting equation 9 with 10 gave a more detailed equation for the mean direct cost of labor:

$$\bar{\;C{\left(P_{i}\right)}_{mob}}=\overset{n}{\underset{c=1}{\sum }}\;\left(\;\bar{t_{c}}\;\cdot \bar{Su_{c}}\right)$$ (11).

Substituting equation 2 with equations 5, 8 and 11, the following equation was obtained to determine the mean direct cost of each procedure^6^:

$$\bar{C\left(P_{i}\right)}=\overset{n}{\underset{k=1}{\sum }}\;\left(\;\bar{q_{k}}\;\cdot \bar{Pu_{k}}\right)+\overset{n}{\underset{k=1}{\sum }}\;\left(\;\bar{qs_{k}}\;\cdot \bar{Psu_{k}}\right)+\overset{n}{\underset{c=1}{\sum }}\;\left(\;\bar{t_{c}}\;\cdot \bar{Su_{c}}\right)$$ (12).

Finally, the mean direct cost of hemodialysis component procedures was obtained from the insertion of the mean values ​​ obtained by equation 12 into equation 1. The Brazilian currency, the real (R$), originally used for the calculations, was converted into (US$), with the rate of US$ 0.45/R$1.00, based on the quotation of 05/31/2014, provided by the Central Bank of Brazil.

The personnel responsible for human resource services were asked to complete the spreadsheet related to the gross salary (basic salary, benefits, bonuses and social charges) of the nursing professionals, by category, and those responsible for the purchasing departments, were asked for the costs related to the latest acquisitions of materials and solutions/medications.

Considering the absence and/or difficulty of access to information, in the three hospitals, which would enabled the calculation of the indirect costs necessary for the composition of the total cost of the procedures under study, the research was restricted to the use of direct costs. 

Direct costs are defined as monetary expenditure applied in the production of products or services where there is the possibility of identification with the product or department. Direct cost is defined as any that can be measured, that is, that can be identified and clearly quantified^7^. The hospital units are basically composed of labor, inputs and equipment used directly in the care process^8^. Direct labor refers to personnel who work directly on the products or services provided, as long as it is possible to measure the time spent and identify who performed the work. It is composed of salaries, social charges, holiday provisions and 13^th^ salary^7^.

## Results

A total of 22 nursing professionals (61.11%) were observed in the dialysis center of hospital A, with a capacity for attending up to 26 chronic kidney patients/period, 13 professionals (30.23%) in the dialysis center of hospital B, with up to eight chronic and acute kidney patients/period, and seven professionals (100%) in the dialysis center of hospital C, with a capacity to attend up to five chronic and acute kidney patients/period.

Among the 185 patients that were observed, all the patients in hospital A had terminal CKD, and the majority (69.66%) had arteriovenous fistula as the access route for the hemodialysis. In hospitals C and B, the majority of the patients had a dual lumen catheter (85.71 and 82.67%, respectively), and patients with terminal CKD and acute kidney injury were observed.

The inputs used for the assembly of the hemodialysis machine were grouped in the procedure “preparation of hemodialysis equipment”, which consisted of new extracorporeal circuits, as established in hospital B, which disposed of them after each use or, as occurred in hospitals A and C, were manually reprocessed for up to 12 times. In the three hospitals, the procedure was performed most of the time by nursing technicians/assistants: 89.74, 87.13 and 52.52%, respectively. A significant participation of nurses was observed in hospital C (47.48%).

In the three hospitals, material cost was the most striking in the composition of the total mean direct cost of the procedure “preparation of hemodialysis equipment”, especially in hospital B (US$24.10; Standard deviation - *sd*=0.07); in which extracorporeal circuits were not reprocessed, followed by the cost of solutions/medications. There was a statistically significant difference in all variables (personnel, material, solutions/medication and total costs) only in hospital A. This information is presented in Table 1.

**Table 1:** Distribution of the observations related to “preparation of hemodialysis equipment” in hospitals A, B and C, according to personnel, material and solutions/medication costs. São Paulo, SP, Brazil, 2014

Observations	Hospital	n	Mean	sd*	Minimum-maximum	95% CI†		P-value^‡^	Post hoc
Personal cost	A	312	0.19	0.12	0.12-0.71	0.17	0.18	<0.001	a
(US$)^§^	B	202	0.25	0.09	0.21-0.47	0.21	0.21		b
	C	100	0.68	0.27	0.38-1.15	0.58	0.78		c
Material cost	A	312	12.47	8.36	3.16-24.41	10.72	14.31	<0.001	a
(US$)^§^	B	202	24.10	0.07	24.03-24.56	24.03	24.03		b
	C	100	10.04	8.72	0.83-37.10	6.78	9.18		c
Solutions/	A	312	9.54	0.39	8.85-9.76	9.76	9.76	<0.001	a
medications costs	B	202	8.25	0.00	8.25-8.25				b
(US$)^§^	C	100	9.58	0.45	9.45-11.16	9.45	9.45		c
Total cost	A	312	22.20	8.06	13.24-34.73	20.60	24.00	<0.001	a
(US$)^§^	B	202	32.60	0.12	32.50-33.00	32.50	32.56		b
	C	100	20.30	8.80	11.10-48.62	16.95	19.58		c

*sd: standard deviation; †CI: confidence interval; ‡: Kruskal-Wallis test; *Post hoc* by the Steel-Dwass-Critchlow-Fligner method, where different letters indicate different distributions; §: conversion rate: US$0.45/R$1.00, based on the quotation of 5/31/2014, provided by the Central Bank of Brazil

In hospital B, the cost of the extracorporeal circuit (first use) corresponded to US$23.26; in hospital A, the cost ranged from US$23.22 (first use) to US$1.94 (12^th^ reuse), and in hospital C, from US$31.10 (first use) to US$3.00 (12^th^ reuse). Regarding the cost of the extracorporeal circuit in the first use, in hospital C this value was 1.34 higher than that of hospitals A and B. The gallons of 8.4% sodium bicarbonate solution and polyelectrolyte solution were the most representative items in the composition of the costs of solutions/medications, especially in hospital C (US$4.00 and US$4.59), followed by hospitals A (US$3.74 and US$4.20), and B (US$3.47 and US$3.82).

The “installation of hemodialysis arteriovenous fistula access” was performed exclusively by nurses in hospital C (100%), predominantly by nurses in hospital B (92.31%) and frequently by nursing technicians (63.09%) in hospital A. Table 2 shows that the cost of material was the most important variable for the mean direct cost of this procedure in hospitals C (US$2.10, *sd*=0.91) and A (US$1.42, *sd*=0.16) and the cost of solutions/medications in hospital B (US$2.20; *sd*=1.89), due to the higher consumption of heparin for blood anticoagulation during extracorporeal circulation. There was a statistically significant difference in the personnel, solutions/medication and total cost variables only in hospital A.

**Table 2:** Distribution of the observations related to “installation of hemodialysis arteriovenous fistula access” in hospitals A, B and C, according to personnel, material and solution/medication costs. São Paulo, SP, Brazil, 2014

Observations	Hospital	n	Mean	sd*	Minimum-maximum	95% CI^†^		P-value^‡^	Post hoc
Personal cost	A	233	0.18	0.08	0.12-0.59	0.18	0.18	<0.001	a
(US$)^§^	B	26	0.46	0.09	0.47-0.69	0.47	0.47		b
	C	25	0.78	0.00	0.78-0.78				c
Material cost	A	233	1.42	0.16	1.28-2.37	1.37	1.41	0.039	a
(US$)^§^	B	26	1.54	0.45	0.89-2.84	1.34	1.70		a
	C	25	2.10	0.91	0.23-2.75	1.93	2.75		b
Solutions/	A	233	0.84	0.34	0.24-2.68	0.73	0.89	<0.001	a
medications costs	B	26	2.20	1.89	0.17-7.72	1.05	2.74		b
(US$)^§^	C	25	0.79	0.36	0.10-1.10	0.60	0.10		a
Total cost	A	233	2.44	0.41	1.68-4.21	2.35	2.44	<0.001	a
(US$)^§^	B	26	4.20	2.0	4,17-10.53	3.11	4.73		b
	C	25	3.67	115	2.00-4.50	3.24	4.50		b

*sd: standard deviation; †CI: confidence interval; ‡: Kruskal-Wallis test; *Post hoc* by the Steel-Dwass-Critchlow-Fligner method, in which different letters indicate different distributions; §: conversion rate: US$0.45/R$1.00, based on the quotation of 5/31/2014, provided by the Central Bank of Brazil

In the majority of the procedures of “installation of hemodialysis dual lumen catheter access”, the participation of the nursing professional was observed (95.80% in hospital B, 81.63% in hospital A and 50.92% in hospital C) and frequently of the nursing technician, corresponding to 99.40% of the observations in hospital B, 98.97% in hospital A and 53.71% in hospital C.

As shown in Table 3, hospital B presented the highest mean cost with solutions/medications (US$2.36, *sd*=0.72), related to the addition of 19.1% potassium chloride ampoules (10 ml ampoule: US$0.14) in the gallon of polyelectrolyte solution. There was a statistically significant difference in all variables (personnel, material, solutions/medication and total costs) only in hospital A.

**Table 3:** Distribution of the observations related to “installation of hemodialysis dual lumen catheter access” in the dialysis centers of hospitals A, B and C, according to personnel, material and solution/medication costs. São Paulo, SP, Brazil, 2014

Observations	Hospital	n	Mean	sd*	Minimum-maximum	95% CI^†^		P-value^‡^	Post hoc
Personal cost	A	98	0.15	0.09	0.12-0.59	0.12	0.12	<.001	a
(US$)^§^	B	167	0.25	0.11	0.21-0.68	0.21	0.21		b
	C	108	0.88	0.35	0.38-1.92	0.77	20.96		c
Material cost	A	98	2.56	2.93	0.63-9.37	1.18	1.71	<0.001	a
(US$)^§^	B	167	1.52	1.07	0.62-9.78	1.26	1.42		a
	C	108	4.41	4.36	0.90-212.28	2.09	6.00		b
Solutions/	A	98	2.07	7.48	0.13-44.64	0.73	0.89	<0.001	a
medications costs	B	167	2.36	0.72	0.12-4.22	5.13	5.57		b
(US$)^§^	C	108	2.20	0.14	1.972.39	2.19	2.23		c
Total cost	A	98	4.78	8.70	0.88-52.22	2.11	2.90	<0.001	a
(US$)^§^	B	167	4.13	1.37	0.95-13.87	3.91	4.17		b
	C	108	749	4.44	3.32-15.80	5.233	9.11		c

*sd: standard deviation; †CI: confidence interval; ‡: Kruskal-Wallis test; *Post hoc* by the Steel-Dwass-Critchlow-Fligner method, in which different letters indicate different distributions; §: conversion rate: US$0.45/R$1.00, based on the quotation of 5/31/2014, provided by the Central Bank of Brazil

In the “installation of hemodialysis dual lumen catheter access” the highest total direct cost was found in hospital C (US$7.49; *sd*=4.44) associated with the high mean cost of material (US$4.41; *sd*=4.36). In this hospital, transparent film dressing was used (unit: US$1.34) at the catheter insertion site, and the addition of new Tego^®^ connectors (US$4.50 per pair) was observed for protection of the catheter routes, as well as the second highest mean cost with solutions/medications consumed, such as benzine (10 ml: US$0.07), to remove the dressing at the catheter insertion site, and mupirocin ointment (10 mg: US$0.27), in addition to the inputs common to hospitals A and B.

The “removal of hemodialysis arteriovenous fistula access” was performed predominantly by nursing technicians in hospitals B and A (84.61 and 81.54%) and by nurses in hospital C (76.00%), which justifies the higher mean personnel cost in this hospital (US$0.72; dp=0,23). There was a statistically significant difference in the components of the procedure variables (personnel, material and total costs) only in hospital A.

The procedure of “removal of hemodialysis dual lumen catheter access” was predominantly performed by nursing technicians in hospitals A and B (98.97% and 94.61%) and nurses in hospital C (60.20%), which, as shown in Table 5, presented the highest mean cost with personnel (US$0.86; *sd*=0.34). In all three hospitals, the procedure was performed by only one nursing professional, in the majority of the observations (95.21% in hospital B, 70.37% in hospital C and 58.16% in hospital A).

**Table 4:** Distribution of the observations related to the “removal of hemodialysis arteriovenous fistula access” in hospitals A, B and C, according to personnel and material costs. São Paulo, SP, Brazil, 2014

Observations	Hospital	n	Mean	sd*	Minimum-maximum	95% CI^†^		P-value^‡^	Post hoc
Personal cost	A	233	0.17	0.07	0.12-0.36	0.17	0.17	<0.001	a
(US$)^‡^	B	26	0.27	0.16	0.21-0.95	0.21	0.21		b
	C	25	0.72	0.23	0.38-1.54	0.58	0.77		c
Material cost	A	233	0.29	0.07	0.26-0,68	0.27	0.27	<0.001	a
(US$)^‡^	B	26	0.27	0.07	0.11-0.40	0.25	0.31		b
	C	25	0.32	0.07	0.16-0.46	0.31	0.31		c
Total cost	A	233	0.46	0.10	0.38-1.03	0.44	0.44	<0.001	a
(US$)^‡^	B	26	0.54	0.19	0.32-1.33	0.46	0.59		a
	C	25	1.04	0.27	0.69-2.00	0.90	1.08		b

*sd: standard deviation; †CI: confidence interval; ‡: Kruskal-Wallis test; Post hoc by the Steel-Dwass-Critchlow-Fligner method, in which different letters indicate different distributions; §: conversion rate: US$0.45/R$1.00, based on the quotation of 5/31/2014, provided by the Central Bank of Brazil

**Table 5:** Distribution of the observations related to “removal of hemodialysis dual lumen catheter access” in the dialysis centers of hospitals A, B and C, according to personnel, material and solutions/medications costs. São Paulo, SP, Brazil, 2014

Observations	Hospital	n	Mean	sd*	Minimum-maximum	95% CI^†^		P-value^‡^	Post hoc
Personal cost	A	98	0.18	0.07	0.12-0.36	0.18	0.18	<0.001	a
(US$)^§^	B	167	0.23	0.07	0.21-0.47	0.21	0.21		b
	C	108	0.86	0.34	0.38-1.54	0.77	0.96		c
Material cost	A	98	0.96	1.11	0.70-11.72	0.82	0.89	<0.001	a
(US$)^§^	B	167	2.36	2.92	0.31-8.18	0.75	2.47		b
	C	108	0.80	0.55	0.32-2.14	0.57	0.72		c
Solutions/	A	98	3.95	7.24	0.13-46.21	2.65	2.96	<.001	a
medications costs	B	167	1.26	1.49	0.00-6.57	1.26	2.08		b
(US$)^§^	C	108	0.90	5.79	0.00-42.83	0.13	0.13		c
Total cost	A	98	5.09	7.34	0.95-47.54	3.65	4.00	<0.001	a
(US$)§	B	167	3.85	3.50	0.53-12.98	2.46	4.28		b
	C	108	2.56	5.75	0.73-43.95	1.58	1.87		c

*sd: standard deviation; †CI: confidence interval; ‡: Kruskal-Wallis test; *Post hoc* by the Steel-Dwass-Critchlow-Fligner method, in which different letters indicate different distributions; §: conversion rate: US$0.45/R$1.00, based on the quotation of 5/31/2014, provided by the Central Bank of Brazil

In hospital A there was the highest mean cost with solutions/medications (US$3.95; *sd*=7.24), associated with the consumption of saline solution (SS 0.9%) and of 10 mg Actilyse (3 ml: US$42.83), for the clearing of the lumen of the catheter. Again, the only hospital that had a statistically significant difference in all variables (personnel, material, solutions/medication and total costs) was hospital A.

The second highest mean cost with solutions/medications in hospital B (US$1.26; *sd*=1.49) was related to the use of SS 0.9% (250 ml bottle: US$0.50) and higher doses of heparin used to fill the lumens of the catheter. In the majority of the observations regarding hospital C (62.04%), gentamicin (ampoule: US$0.13) was administered in the “removal of hemodialysis dual lumen catheter access”. Hospital B had the highest material mean cost of material (US$2.36; *sd*=2.92) associated with the consumption of Tego^®^ connectors (pair: US$3.60).

Among the patients with an arteriovenous fistula, mostly in hospital A (69.66%), the total mean direct cost of the procedures studied corresponded to US$25.10 in hospital A, US$37.34 in hospital B and US$25.01 in hospital C. In patients with a dual lumen catheter, mostly in hospitals C (85.71%) and B (82.67%), the total mean direct cost was US$30.35 in hospital C, US$3.07 in hospital A and US$40.58 in hospital B. The weighted means of these values ​​resulted in US$26.59 for hospital A, US$38.96 for hospital B and US$27.68 for hospital C.

## Discussion

In the procedures studied, the participation of nurses and nursing technicians was influenced by the care capacity, physical area of ​​the dialysis centers and the relationship between the number of nursing professionals, by category, and the quantitative and clinical conditions of the patients with CKD (in the three hospitals) or acute renal injury (in hospitals B and C). The nurses took responsibility for the performance of procedures for patients with a more complex profile, especially when there was a problem related to the venous access.

The arteriovenous fistula was the predominant method of access among the patients of hospital A (69.66%) and the dual lumen catheter among the patients of hospitals C and B (85.71 and 82.67%). The indication of arteriovenous fistula has proven to be of great benefit to patients undergoing hemodialysis^9^, with catheter use being associated with a higher incidence of infections, hospitalizations, and increased patient mortality^10^.

It was found that the procedures of “installation and removal of hemodialysis arteriovenous fistula access” presented a lower economic impact on the total mean direct cost compared to the procedures “installation and removal of hemodialysis dual lumen catheter access”. In view of these results, apart from the humanitarian, ethical, scientific and political aspects, the economic aspect should be considered, since the adoption of the arteriovenous fistula is indicates as the preferential venous access for hemodialysis.

Material costs, especially extracorporeal circuits, and medication/solutions corresponded to the values ​​that contributed the most to the total mean direct cost, similarly to the results of other studies on the direct costs of procedures performed by nursing professionals^11^ ^-^ ^12^.

With regard to the extracorporeal circuits, the Brazilian Health Regulatory Agency (ANVISA), through the Resolution of the Board of Directors (RDC) No. 11/2014, among other provisions, prohibits the reuse of dialysers that are labeled with “reprocessing prohibited”, the reuse of dialysers that do not have biocompatible membrane capillaries and the reuse of arterial and venous lines that should be discarded after use. Therefore, until the deadline for compliance (three years from the date of publication of the CDR), the arterial and venous lines should be considered together with the dialysers (for control of the reuse and disposal) submitted to manual processing and should be used for the same patient for a maximum of 12 times^13^.

If the dialysis centers of hospitals A and C had already adopted the provisions of RDC No.11/2014^13^ and did not reprocess the extracorporeal circuits, the total mean direct cost of “preparation of the hemodialysis equipment” would be US$34.00 (*sd*=0.42) and US$47.43 (*sd*=0.61) respectively, corresponding to an increase of US$11.80 in hospital A and US$27.13 in hospital C.

It is emphasized that when the organization is able to purchase larger volumes there is a greater possibilities of negotiation to reach the lowest price. The lower prices will depend on the trading skills of the purchasing professionals, who should have access to the technical recommendations of the inputs, know the market trends, evaluate the existence of competitive products and have computerized and updated databases available for obtaining information on the historical trends of prices and purchasing volumes. From this perspective, the best performance of these professionals will depend on the advice of the technicians responsible in specific areas, such as the dialysis centers, for the proper preparation of the bidding documents and the contracts for the bidding process^6^.

Bringing together the consumer sectors and the administration and purchasing sectors, through technical advisory services and commissions composed of doctors, nurses, pharmacists and physiotherapists, among others, who, based on their technical knowledge, contribute to the decision-making regarding the adoption, discontinuity and minimum quality requirements of the inputs used, constitutes an efficient proposal to improve the management of materials in hospitals^14^.

The high cost of material resources has intensified the concern of health organization managers who need to invest in the improvement of material management systems in order to provide continuity of quality care at a lower cost by ensuring adequate quantity and quality^15^.

It is known that hospitals are involved in a very complex economic environment, with technical and technological updates being necessary to ensure the quality and competence in the provision of services. The demand for services is also increasing and the lack of resources, for both SUS and health insurance operators, aggravates their financial situation^16^.

Thus, the rapid increase in costs to meet the different care demands in hospital organizations requires studies on the financial aspects involved, which will make it possible to propose strategies for the efficient use of resources, balancing the provision of health services and their economic viability^17^. 

Hospital organizations undoubtedly need detailed and consistent information on the costs incurred in the provision of their services, since they assist in the adequate application of scarce resources, especially in hospitals providing services to the SUS^12^. However, efforts need to be centered beyond cost containment strategies, since it is necessary to know how these cost are formed in the different care processes, in order to improve the distribution of resources and services without losing quality, increasing the accessibility for the users^18^.

The comparison of the costs of “preparation of hemodialysis equipment” between two dialysis centers that reprocessed the extracorporeal circuits (A and C) and one that did not (B), due to being within the prescribed period for compliance with RDC No. 11/2014^13^, can be considered a limitation of this study. However, because in the national scenario, there is little research in which the costs of procedures, especially those related to dialysis, have been calculated, this study represents an initial and unpublished approach, in which a methodology is proposed that can be reproduced and will enable the calculation and management of the direct costs of constituent procedures of conventional hemodialysis.

As implications for professional practice, it is hoped that the analysis of the direct costs of the integrated hemodialysis procedures, by adding knowledge to this thematic area, supports hospital managers, technicians responsible and nursing professionals in detecting inefficiencies and waste, as well as intervening in the productive process for the rational allocation of the inputs available in the dialysis centers.

## Conclusion

The weighted means of the direct mean costs of the procedures under study corresponded to US$26.59 for hospital A, US$38.96 for hospital B and US$27.68 for hospital C. In the three hospitals, material and medication/solution costs were the ones that contributed the most to the total mean direct cost per procedure.

The procedures for the “installation and removal of hemodialysis arteriovenous fistula access” (US$25.10 in hospital A, US$37.34 in hospital B and US$25.01 in hospital C) had a significantly lower financial impact compared to procedures for the “installation and removal of hemodialysis dual lumen catheter access” (US$32.07 in hospital A, US$40.58 in hospital B and US$30.35 in hospital C), reiterating the preference for arteriovenous fistula as an economically favorable venous access for this type of renal replacement therapy.
